# Supplementary material for: A general model to predict small molecule substrates of enzymes based on machine and deep learning
Source: Nat Commun. 2023 May 15;14:2787. doi: 10.1038/s41467-023-38347-2 (PMC10185530; doi:10.1038/s41467-023-38347-2)
Supplement: Supplementary file 1 — Supplementary Information [file 41467_2023_38347_MOESM1_ESM.pdf]

A general model to predict small molecule substrates of enzymes based on machine and deep learning

Alexander Kroll, Sahasra Ranjan, Martin K. M. Engqvist, Martin J. Lercher

This PDF includes:

Supplementary Tables 1-5

Supplementary Figures 1-5

Supplementary Tables 1-5

Supplementary Table 1. Results of hyperparameter optimizations of the gradient boosting models for all four combinations of small molecule representations (ECFPs and GNN generated fingerprints) and enzyme representations (*ESM-1b* and *ESM-1b*$_{ts}$ vectors). The hyperparameter optimizations were performed with 5-fold cross-validation on the training set.

	**mean ROC-AUC (CV)**	**learning rate**	**max. delta step**	**max. depth**	**min. child weight**	**num. of trees**	**alpha coeff.**	**beta coeff.**	**weight**
***ESM-1b* & ECFP**	0.861	0.127	3.08	13	2.69	333	1.43	0.12	0.114
***ESM-1b*$_{ts}$ & ECFP**	0.911	0.316	1.77	10	1.38	343	0.53	3.74	0.262
***ESM-1b* & GNN**	0.888	0.081	4.90	11	4.48	347	0.35	0.62	0.127
***ESM-1b*$_{ts}$ & GNN**	0.926	0.198	3.82	12	0.96	358	0.37	4.44	0.113

Supplementary Table 2. Results of three different machine learning algorithms on the test set. Hyperparameter optimizations for all models were performed with 5-fold cross-validation on the training set.

	**ROC-AUC score**	**Accuracy**	**MCC**
**Gradient Boosting**	0.955	91.5%	0.78
**Random Forest**	0.945	87.7%	0.67
**Logistic Regression**	0.621	63.0%	0.14

Supplementary Table 3. Results of a gradient boosting model with $ESM\text{-}1b_{ts}$ vectors and GNN-generated fingerprints (with a pre-trained GNN) compared to a gradient boosting model with $ESM\text{-}1b_{ts}$ vectors and GNN-generated fingerprints (with a not pre-trained GNN). Results are shown for the test set. The hyperparameter optimizations for all models were performed with 5-fold cross-validation on the training set.

	**ROC-AUC score**	**Accuracy**	**MCC**
**GNN-generated fingerprints with pre-trained GNN**	0.955	91.5%	0.78
**GNN-generated fingerprints with not pre-trained GNN**	0.954	90.7%	0.77

Supplementary Table 4. Results of three gradient boosting models with different enzyme representations. Models were trained with GNN-generated fingerprints as small molecule representations combined with three different enzyme representations: *ESM-1b* vectors, *ESM-1b*$_{ts}$ vectors created without an extra token for the whole enzyme, and *ESM-1b*$_{ts}$ vectors created with an extra token for the whole enzyme. Results are shown for the test set. The hyperparameter optimizations for all models were performed with 5-fold cross-validation on the training set.

	**ROC-AUC score**	**Accuracy**	**MCC**
ESM-1b	0.940	88.8%	0.72
***ESM-1b*$_{ts}$ (mean representation)**	0.956	90.9%	0.77
***ESM-1b*$_{ts}$ (enzyme token)**	0.956	91.5%	0.78

Supplementary Table 5. Results of validating the ESP model on the test sets from Yang et al.[1] and Mou et al.[2] without adding any new training data to our training set.

	**ROC-AUC score**	**Accuracy**	**MCC**
**Yang et al.** ***Avena strigosa***	0.59	68%	0.12
**Yang et al.** ***Lycium barbarum***	0.56	66%	0.01
**Mou et al.**	0.41	0.5%	0.00

Supplementary Figures 1-5

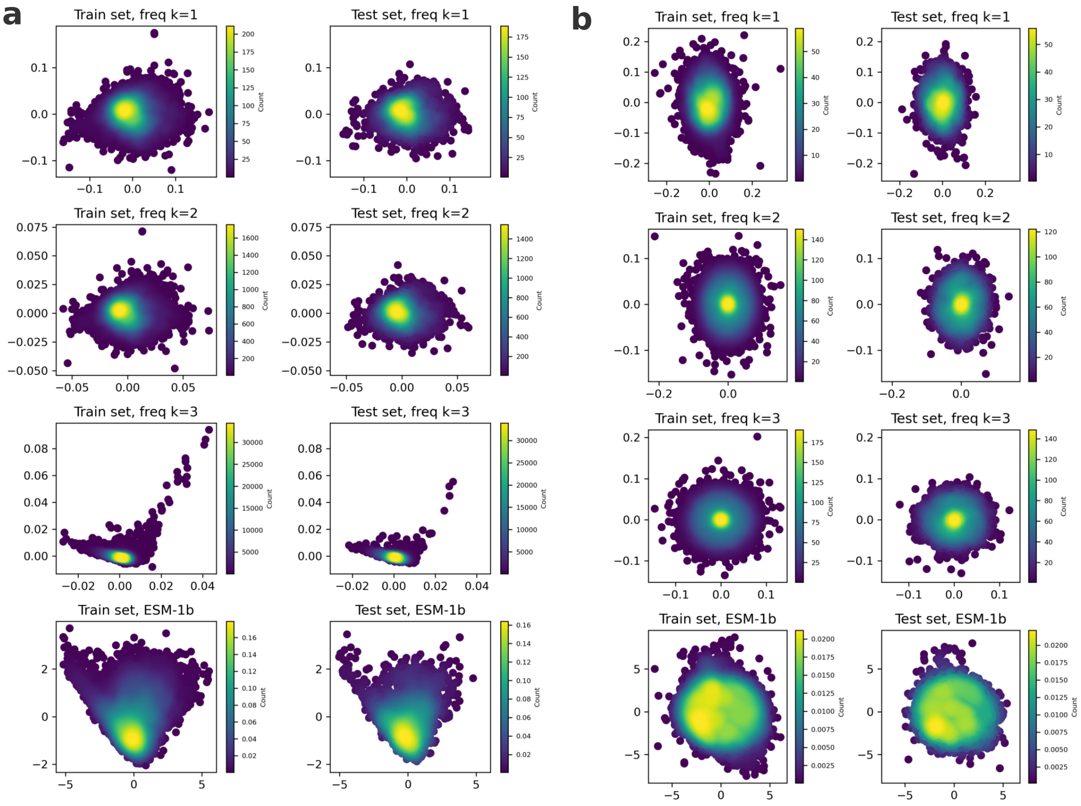

Supplementary Figure 1. Similar distributions of enzymes in the training and the test sets. We projected numerical representations of the enzyme amino acid sequences to two-dimensional spaces, using two different types of representations: (i) We created vectors with the frequencies of specific k-mers of amino acids within the protein amino sequences (for $k = 1, 2, 3$) for all enzymes in the training and the test set; and (ii) we used the *ESM-1b* vectors. **(a)** Projection onto the first two principal components after Principal Component Analysis (PCA). **(b)** Multidimensional scaling (MDS) onto a two-dimensional space.

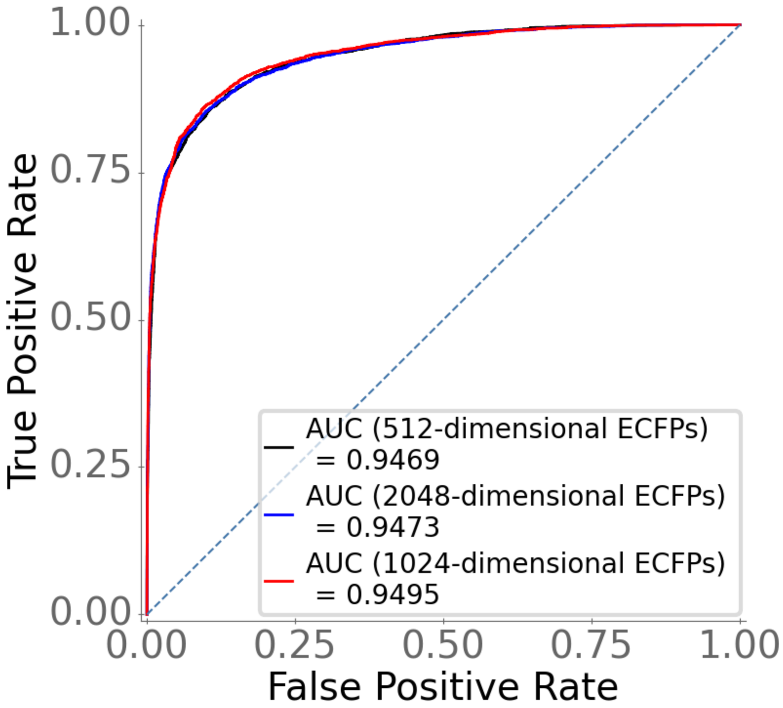

Supplementary Figure 2. Effect of different dimensions of ECFPs. We calculated extended-connectivity fingerprints (ECFPs) as representations for small molecules using different dimensions, comparing 512-, 1 024-, and 2 048-dimensional ECFPs. The plot shows ROC curves resulting from optimized gradient boosting models. For training these models, ECFPs of different dimensions were combined with $ESM\text{-}1b_{ts}$ vectors as enzyme representations.

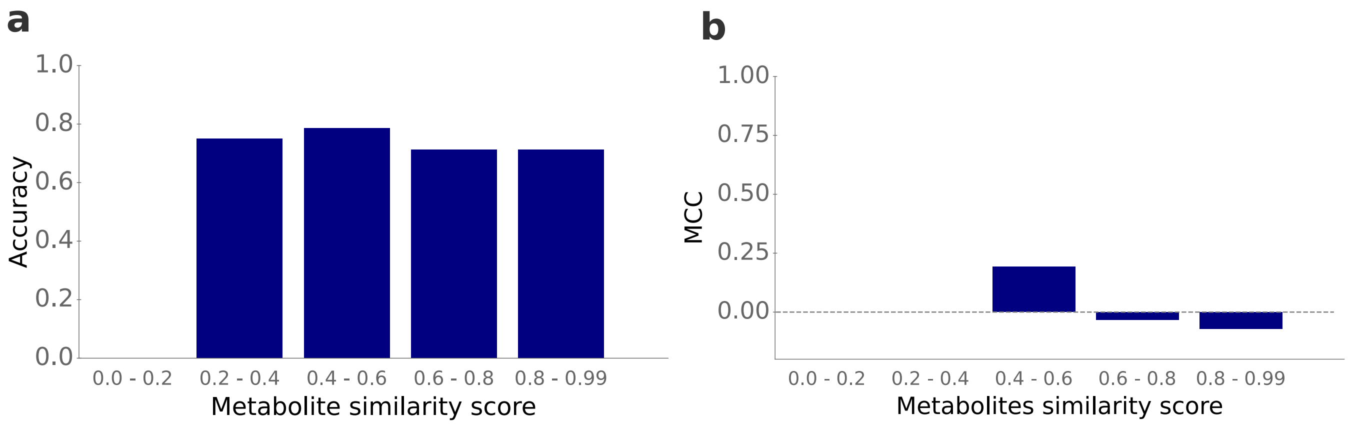

Supplementary Figure 3. Effect of the metabolite similarity score on model performance. For all small molecules in the test set that do not also occur in the training set, we calculated the maximal pairwise similarity score across all small molecules in the training set. The similarity score is a value between 0 and 1, where a higher value indicates higher similarity between a pair of metabolites. We divided all test data points with small molecules that do not occur in the training set into five subsets dependent on their maximal similarity scores. **(a)** shows the accuracy and **(b)** shows the MCC for each subset.

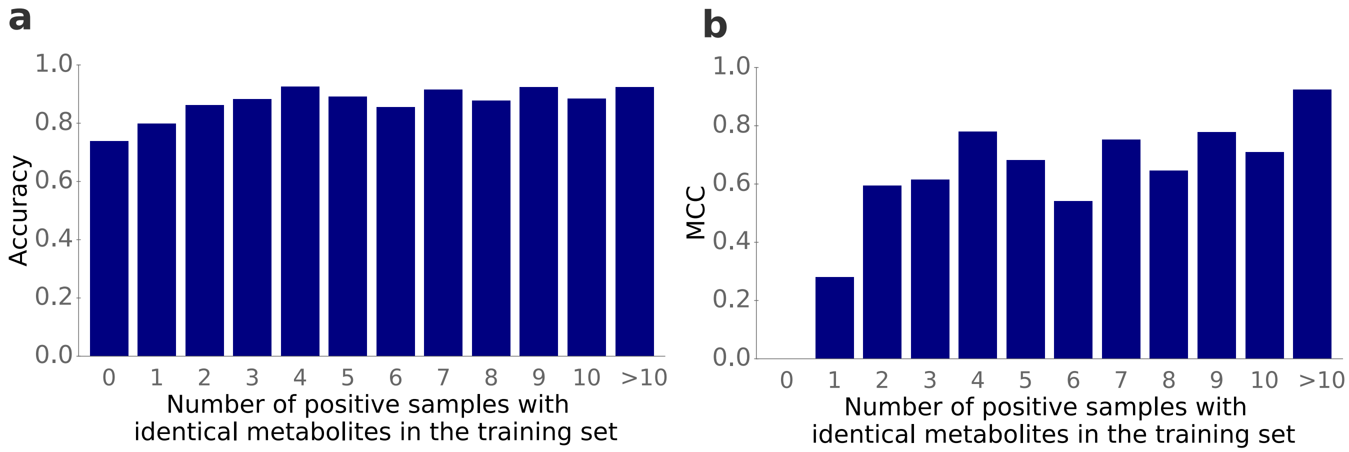

Supplementary Figure 4. Effect of the number of identical substrates in the training set on model performance. We grouped small molecules by how often they occur as substrates among all positive data points in the training set. **(a)** shows the accuracy and **(b)** shows the MCC for each group.

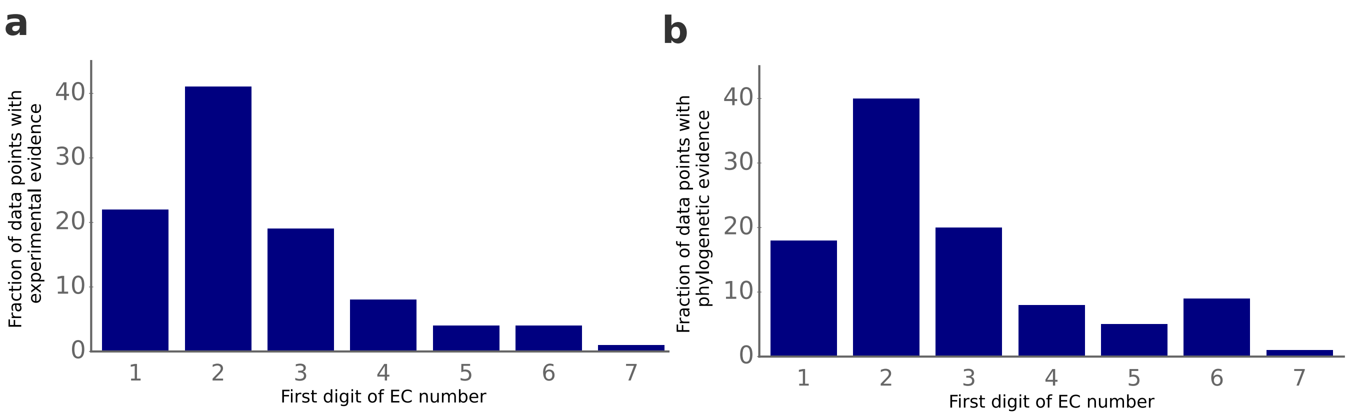

Supplementary Figure 5. Enzymes with experimental evidence and with phylogenetic evidence do not differ strongly in their distribution across top level Enzyme Commission (EC) numbers. (a) Distribution across the first digit of EC numbers for all enzyme-substrate pairs with experimental evidence. (**b**) Distribution across the first digit of EC numbers for all enzyme-substrate pairs with phylogenetically inferred evidence.

Supplementary References

1. Yang, M. *et al.* Functional and informatics analysis enables glycosyltransferase activity prediction. *Nat. Chem. Biol.* **14,** 1109–1117 (2018).
2. Mou, Z. *et al.* Machine learning-based prediction of enzyme substrate scope: Application to bacterial nitrilases. *Proteins Struct. Funct. Bioinf* **89,** 336–347 (2021).
